# Role of hydroxychloroquine in primary glomerular disease - a systematic review and meta-analysis of the current evidence

**DOI:** 10.1186/s12882-025-04370-2

**Published:** 2025-08-04

**Authors:** Gerry George Mathew, Shanmugam Sundaramurthy, Prakash Muthuperumal, V. Jayaprakash

**Affiliations:** 1 Department of Nephrology, SRM Medical College Hospital and Research Centre, Kattankulathur, Tamil Nadu 603203 India; 2 Department of Computing Technologies, SRM Institute of Science and Technology, Kattankulathur, Tamil Nadu 603203 India; 3 School of Public Health, Division of Health Data Science, SRM Institute of Science and Technology, Kattankulathur, Tamil Nadu 603203 India

**Keywords:** Efficacy, eGFR, Hydroxychloroquine, Primary glomerular disease, Proteinuria, Safety

## Abstract

**Background:**

Hydroxychloroquine is increasingly being used to treat primary glomerular diseases. It has shown promising results in terms of reducing proteinuria and stabilizing kidney function. This systematic review aimed to assess the effects of HCQ on proteinuria and the estimated glomerular filtration rate (eGFR) in primary glomerular diseases and evaluate its safety profile.

**Methods:**

A literature search was conducted using PubMed, ScienceDirect, Springer, and Google Scholar for articles published between 2014 and 2024. Articles incorporating hydroxychloroquine for the treatment of primary glomerular diseases were considered. These studies evaluated the effect of HCQ on 24-hour proteinuria and eGFR. Pooled mean differences (MDs) and heterogeneity metrics (Tau², I², and Q-test) were analysed. The safety data from all included studies were reviewed.

**Results:**

HCQ administration significantly reduced proteinuria (MD = -0.69, 95% CI= -0.79 to -0.59), with pronounced effects for longer treatment durations (MD = -0.74, 95% CI= -0.81 to -0.67), and in patients with membranous nephropathy (MD = -3.00, 95% CI= -4.46 to -1.53). Conversely, no significant improvement in eGFR was observed after HCQ treatment (MD = -1.03, 95% CI= -2.73 to − 0.67). A beneficial effect was noticed in patients with IgA nephropathy (MD = -2.65, 95% CI= -5.16 to -0.14). Moderate to substantial heterogeneity (I² = 69–95%) was observed for proteinuria outcomes, but no heterogeneity was found for eGFR outcomes (I² = 0%). 69 adverse events were reported, of which gastrointestinal and mucocutaneous effects were the most common.

**Conclusion:**

HCQ therapy for primary glomerular diseases showed a trend toward reducing proteinuria without significant effects on eGFR, with a better safety profile.

**Trial registration:**

This systematic review was registered in PROSPERO with ID CRD42024597762.

## Introduction

Glomerular diseases are renal conditions that affect glomerular capillaries or the glomerular basement membrane [1]. This damage often occurs because of inflammation/involvement of any of the three components (capillary endothelium, mesangium, or basement membrane) of the glomeruli [2]. Such an affliction may occur directly in the kidneys without a known external factor and may result from a combination of immune, genetic, or environmental factors, categorized as primary glomerular diseases. Examples of primary glomerular diseases include minimal change disease (MCD), focal segmental glomerulosclerosis (FSGS), membranous nephropathy (MN), IgA nephropathy and idiopathic membranoproliferative glomerulonephritis (MPGN) [1]. Secondary glomerular diseases, on the other hand, are caused by systemic conditions such as diabetes, lupus, certain infections, medications, or malignancies that affect the kidney. Common examples of secondary glomerulopathies include diabetic nephropathy, lupus nephritis, and infection-related glomerulonephritis. Understanding the type and cause of glomerular diseases is crucial for determining the appropriate treatment and management strategies.

Glomerular diseases can present with a wide range of symptoms. Some patients may be asymptomatic, and some present with haematuria, while others develop sub-nephrotic proteinuria or nephrotic syndrome [3]. These diseases contribute significantly to the increasing prevalence of chronic kidney disease (CKD) [4]. Glomerular diseases are a prominent cause of end-stage renal disease (ESRD), which often necessitates dialysis or kidney transplantation and creates a heavy financial and medical burden on healthcare systems [5, 6]. Owing to increasing CKD rates, it is vital for healthcare providers to look for effective management strategies. Glomerular diseases are primarily treated with immunosuppressive agents such as rituximab, calcineurin inhibitors, mycophenolate mofetil, cyclophosphamide, and corticosteroids [2]. While these medications are effective in controlling disease progression [7, 8], they are also associated with serious long-term side effects, particularly cardiovascular complications, such as myocardial infarction, infections, neoplasia, and cerebrovascular accidents [9, 10]. Additionally, long-term use of these drugs can lead to resistance and loss of efficacy, making it necessary to carefully balance treatment benefits and potential risks. This highlights the importance of exploring alternative therapies to improve outcomes and minimize the associated adverse effects in patients with glomerular diseases, along with low-dose traditional immunosuppressants.

Hydroxychloroquine (HCQ), a 4-aminoquinolone originally developed as an antimalarial agent, has emerged as an effective immunomodulator of autoimmune diseases [11]. Scientific exploration has led to the identification of its mechanism of action involving interference with lysosomal activity, inhibition of autophagy, reduction in the number of activated immune cells, including Toll-like receptor (TLR)-expressing cells and interferon (IFN)-secreting dendritic cells, decrease in the production of pro-inflammatory cytokines such as IL-6, IFN-α, and TNF- α, and modulation of antigen presentation through inhibition of the major histocompatibility complex (MHC)-II pathway [12]. Its immunomodulatory, antithrombotic, and anti- inflammatory properties have led to its use in managing systemic lupus erythematosus (SLE) and rheumatoid arthritis [13]. In SLE, HCQ contributes significantly to improving remission rates and other clinical outcomes, such as enhancing pregnancy results, preserving bone morphology and architecture, and decreasing the likelihood of complications resulting from thrombosis and heart-related issues [14].

Given its versatility, HCQ has been used for other clinical conditions, including primary glomerular diseases. Studies suggest that HCQ may improve renal remission and protect kidneys against severe damage, particularly in lupus nephritis [15]. It was further observed that the likelihood of developing renal failure could be reduced if this antimalarial drug was administered prior to the onset of lupus nephritis [16]. For IgA nephropathy, HCQ has demonstrated its ability to reduce pathogenic IgA production [17] and lower proteinuria levels on both short- and long-term bases without serious side effects [18, 19]. Moreover, in cases where corticosteroids are not tolerated, HCQ offers a safe and effective treatment option [20], as highlighted by the 2021 Kidney Disease Improving Global Outcomes (KDIGO) Clinical Practice Guidelines, which recommend HCQ for IgA nephropathy alongside supportive care [21]. In primary membranous nephropathy, HCQ has also shown antiproteinuric properties, particularly in PLA2R-associated diseases [22]. In their study, patients treated with HCQ achieved a > 30% reduction in proteinuria after six months and clinical remission after two years, along with significant reductions in anti-PLA2R antibody levels. This suggests that HCQ’s immunomodulatory effects of HCQ contribute to its therapeutic benefits [23]. Additionally, a case study described the successful treatment of membranous nephropathy with concomitant diabetic nephropathy using HCQ and rituximab, although HCQ’s specific contribution of HCQ in this clinical scenario remains unclear [24]. Ongoing research aims to further explore the efficacy of HCQ in primary membranous nephropathy and other primary glomerular diseases, highlighting its potential as a therapeutic option in this challenging group of disorders.

### Objective of the study

Hydroxychloroquine (HCQ), with its immunomodulatory and anti-inflammatory properties, has shown promise in reducing proteinuria and stabilizing kidney function in some glomerular diseases [12]. Most available studies have focused on HCQ’s role in secondary glomerular diseases, such as lupus nephritis [15, 25], leaving its potential for primary glomerular diseases underexplored [26, 27]. Furthermore, there is a lack of data on how HCQ’s effects vary based on disease subtypes, patient demographics, and treatment duration. Addressing these gaps is critical for evaluating HCQ as a viable therapeutic alternative for primary glomerular diseases and for reducing the dependency on traditional immunosuppressive therapies with high side-effect profiles.

Therefore, this study aimed to systematically evaluate existing evidence on the efficacy and safety of HCQ in managing primary glomerular diseases. Its efficacy will be assessed by analysing its impact on clinical outcomes, including proteinuria, estimated glomerular filtration rate (eGFR), remission rates, and relapse rates. The safety of HCQ therapy was also evaluated by determining the incidence rate and types of adverse effects experienced by patients with primary glomerular diseases. This comprehensive assessment aids in understanding the therapeutic potential and risk profile of HCQ in primary glomerular diseases and identifying gaps in current knowledge to guide future research directions.

### Methodology

The guidelines outlined in the Preferred Reporting Items for Systematic Reviews and Meta-Analyses (PRISMA) were adhered to in this systematic review and meta-analysis. This systematic review is registered in PROSPERO with ID: CRD42024597762.

### PICO framework

The Population, Intervention, Comparison/Control, and Outcome (PICO) framework was used to formulate the following research question:

What is the efficacy and safety of hydroxychloroquine for treating patients with primary glomerular diseases?

For this study, the population included adult patients aged 18 years or older diagnosed with primary glomerular disease. The intervention of interest was oral administration of hydroxychloroquine. The control group was the population that received the standard of care/placebo, which was devoid of the additional molecule hydroxychloroquine. The literature review focused on various clinical outcomes and adverse effects, as reported across randomized and non-randomized studies.

### Selection criteria

The inclusion criteria for the study were as follows: (1) studies involving patients diagnosed with primary glomerular disease; (2) studies incorporating hydroxychloroquine as a part of the treatment regimen for affected patients with doses ranging from to 200–400 mg or 5 mg/kg body weight; (3) primary glomerular diseases considered for the study, including minimal change disease, focal segmental glomerulosclerosis, membranous nephropathy, IgA nephropathy, and idiopathic membranoproliferative glomerulonephritis; (4) publications available in English; and (5) studies published in peer-reviewed journals between 2014 and 2024.

The exclusion criteria were as follows: (1) systematic reviews, case reports, conference abstracts, posters, and book chapters; (2) studies focusing on the use of hydroxychloroquine for systemic lupus erythematosus and lupus nephritis; (3) studies using hydroxychloroquine for treating rheumatological conditions; (4) studies reporting hypersensitivity to hydroxychloroquine; and (5) studies published in languages other than English.

### Search strategy

The search was conducted using keywords identified from prior research on hydroxychloroquine in primary glomerular diseases. These terms included “hydroxychloroquine,” “chloroquine,” “plaquenil,” “primary,” “idiopathic,” “membranous nephropathy,” “glomerulonephritis, membranous,” “membranous glomerulopathy,” “membranous glomerulonephritis,” “glomerulonephritis, IgA,” “IgA nephropathy,” “glomerulonephritides IgA,” “IgA glomerulonephritis,” “immunoglobulin A nephropathy,” “Berger’s disease”; “IgA type nephritis,” “minimal change disease,” “glomerulosclerosis, focal segmental,” “focal segmental glomerulosclerosis,” “glomerulonephritis, membranoproliferative,” “membranoproliferative glomerulonephritis.” A literature search was conducted across five electronic databases: PubMed, ScienceDirect, Wiley Online Library, Springer, and Google Scholar. Logical combinations of appropriate keywords and Medical Subject Headings (MeSH) terms were used to conduct the search. Manual searches within the references of selected studies and review articles further supplemented the search.

Only human studies published in peer-reviewed journals in English between January 2014 and October 2024 were included. Table 1 provides comprehensive details on the electronic databases, search terms, filters, and the number of retrieved results.

**Fig. 1:**
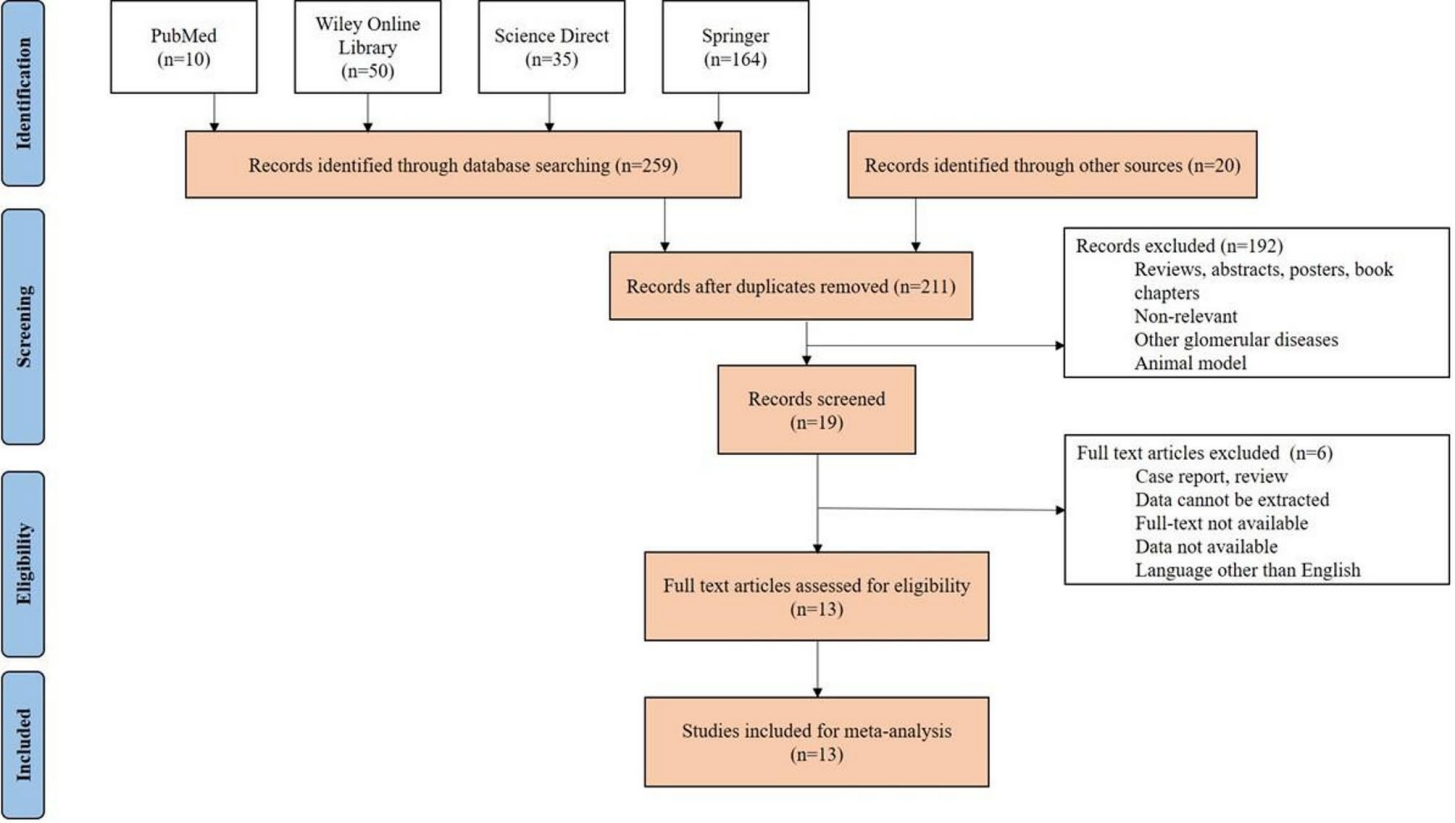
Literature review based on PRISMA protocol

Following PRISMA guidelines, the study selection, evaluation, and synthesis processes were meticulously documented. Figure 1 illustrates a flowchart of the review process. A total of 279 articles were retrieved, with contributions from PubMed (10 articles), the Wiley Online Library (50), ScienceDirect (35), Springer (164), and Google Scholar (20). After removing 68 duplicate records, 192 articles were excluded due to irrelevance, review-type formats, or focus on conditions such as systemic lupus erythematosus, lupus nephritis, or rheumatological disorders.

Nineteen studies underwent manual screening, and 13 full-text articles that satisfied all the eligibility criteria were ultimately included in the meta-analysis.

### Study selection and data screening process

The study selection involved a systematic search across multiple databases, including PubMed, Wiley Online Library, ScienceDirect, Springer, and Google Scholar, using prespecified search terms. Initially, the titles and abstracts of the retrieved articles were screened to exclude irrelevant studies that did not address the research question. Duplicate entries and redundant publications were removed. Full-text versions of shortlisted articles were then obtained and evaluated against a set of predefined inclusion and exclusion criteria. To facilitate organization and subsequent analysis, the references of the selected studies were systematically documented in an Excel spreadsheet.

### Data extraction

Relevant data were extracted from each included study, covering various aspects, such as study details (first author’s name, year of publication, study design, and country of origin), patient baseline characteristics, diagnosed type of glomerular disease, treatment regimens, treatment duration, patient outcomes, and any adverse effects of the interventions. Specific baseline patient characteristics included the total number of participants, sex distribution, age, baseline values for 24-h proteinuria, serum albumin, serum creatinine, and estimated glomerular filtration rate before treatment. Following this extraction process, data were subjected to quantitative analysis.

### Types of outcome measures and their definitions

This study evaluated the efficacy and safety of hydroxychloroquine in adult patients with primary glomerular disease using various outcome measures. Primary outcomes included 24-h proteinuria, estimated glomerular filtration rate, remission (complete and/or partial) rates, relapse rate, and treatment-associated adverse effects. As per definitions, complete remission (CR) was referred to as achieving a proteinuria level of 0.5 g/24-h or less. Partial remission (PR) was defined as a reduction in proteinuria of at least 50% from baseline, with a final proteinuria level between 0.5 and 3.5 g/24-h. Relapse was defined as the reappearance of the proteinuria level above 3.5 g/24-h following CR or PR or presence of active urinary sediments /increase in proteinuria greater than 50% after complete remission in case of IgA nephropathy.

### Quality and risk of bias assessment

The quality and potential biases of the non-randomized studies were assessed using the Newcastle-Ottawa Scale (NOS), as detailed by Wells et al. [28]. This scale evaluates studies across three domains: selection (four items), comparability (two items), and exposure or outcome (three items). Studies were scored on a scale of 0 to 9 and categorized as low-quality (0–2 points), intermediate-quality (3–5 points), or high-quality (6–9 points). For randomized studies, the Cochrane risk-of-bias tool was used, which includes five domains to determine whether the studies were categorized as having a low or “low risk” or “high risk” of bias.

### Statistical analysis

The collected data were analysed using RevMan software (v5.4). The mean difference (MD) and corresponding 95% confidence intervals (CIs) were calculated for the individual and pooled statistics. Subgroup analyses were performed based on treatment duration (less than 6 months vs. 6 months or more) and disease type (IgA nephropathy vs. membranous nephropathy). Both fixed-effects and random-effects models were used to account for heterogeneity. When the studies had high or extreme heterogeneity, a random-effects model was used for statistical analysis. No or moderate heterogeneity was treated using a fixed- effects model. Heterogeneity was quantified using the *I*^*2*^ statistic, with *I*^*2*^ values of 25%, 50%, and 75% representing low, moderate, and substantial heterogeneity, respectively. Statistical significance was set at a probability value of < 0.05.

## Results and discussion

### Characteristics of included studies

Thirteen studies identified from the literature search were found to be appropriate and included in this meta-analysis (Table 1). Various study designs were used, including retrospective cohort studies, prospective case-control studies, randomized controlled trials (both open-label and double-blind), and single-blind trials. This diversity provides a combination of observational and interventional data. Most of the studies (*n* = 12) were conducted in China, with one study conducted in India (Bagchi, 2022). This indicates a regional focus, predominantly on Asia. All studies were published between 2014 and 2024, with the maximum number of papers published in 2024 (*n* = 4), followed by 2022 (*n* = 3) and 2019 (*n* = 2). The proportion of male participants showed significant variation across studies, with the majority reporting a male percentage exceeding 50%. Regarding age, the adult patient population spanned a broad range, from 30.8 ± 12.2 years to 54.9 ± 13.7 years. Baseline proteinuria levels ranged from 0.68 g/day to 3.38 g/day, with notable variability between the study groups. Estimated glomerular filtration rates (eGFR) varied considerably between studies, ranging from as low as 47.65 mL/min/1.73 m² to 97.41 mL/min/1.73 m², reflecting diverse patient populations with varying levels of kidney function. Overall, the studies included in this meta-analysis demonstrated heterogeneity in study design, population demographics, and baseline characteristics.

**Table 1:** Baseline characteristics of included studies

References	Year	Country	Study design	Gender (M %)	Age (years)	Proteinuria (g/day)	Serum albumin (g/dL)	Serum creatinine (micromole/L)	eGFR (ml/min/ 1.73 m2)
Bagchi et al. [29]	2022	India	retrospective cohort study	62.2	35.1 ± 9.1	2.1 ± 0.8	3.9 ± 0.3	123.79 ± 35.37	74.7 ± 27.4
Gao et al. [30]	2017	China	prospective, paired case–control	35.7	39 ± 9	0.9 ± 0.4	N/A	87 ± 20	83 ± 18
				35.7	41 ± 12	0.7 ± 0.2	N/A	84 ± 19	84 ± 19
He et al. [31]	2024	China	retrospective cohort study	52.63	39.26 ± 11.84	0.89	3.913 ± 0.424	N/A	74.83 ± 14.14
				56	39.48 ± 12.07	0.77	3.979 ± 0.689	N/A	79.26 ± 17.22
Liu et al. [18]	2019	China	Double-blind, randomized, placebo-controlled, phase 2 clinical trial	63	37.6 ± 11.6	1.6	4.16 ± 0.29	N/A	52.1 ± 19.7
				67	35.6 ± 9.6	1.9	4.16 ± 0.38	N/A	55.5 ± 18.7
Liu et al. [32]	2022	China	retrospective case-control study			0.81	N/A	74.20	91.07
						0.68	N/A	75.95	97.41
Mei et al. [22]	2024	China	open label, randomized, controlled study	56	51.5 ± 12.2	3.38	N/A	117.0 ± 36.9	80.7 ± 12.5
				71.1	50.9 ± 13.1	3.23	N/A	121.0 ± 38.1	79.2 ± 7.2
Si et al. [20]	2023	China	retrospective case‒control study	56.4	35.1 ± 9.1	1.72	N/A	N/A	68.54 ± 24.86
				58.9	37.5 ± 14.1	1.86	N/A	N/A	68.37 ± 21.02
Tang et al. [33]	2020	China	retrospective case-control study	34.6	28.8 ± 10.2	2.35	N/A	N/A	47.65
				38.5	30.8 ± 12.2	2.35	N/A	N/A	51.59
Tang et al. [19]	2022	China	retrospective cohort	47.8	39.9 ± 10.1	1.69	N/A	N/A	65.82 ± 25.22
Yang et al. [34]	2018	China	retrospective study	48.9	37.3 ± 8.9	1.5	N/A	135.7 ± 49.4	51.2 ± 21.7
				47.8	37.7 ± 10.8	1.5	N/A	131.2 ± 56.9	51.7 ± 18.9
Yang et al. [35]	2019	China	retrospective, case-control	50	37.0 ± 10.0	1.7	N/A	119.8 ± 37.7	56.8 ± 20.4
				50	37.2 ± 12.6	1.8	N/A	127.6 ± 53.8	55.2 ± 22.9
Yang et al. [36]	2024	China	open-label, prospective study	80.6	54.9 ± 13.7	7.6	2.25 ± 0.39	N/A	93.8 ± 17.0
				63.4	53.3 ± 9.5	7.1	2.19 ± 0.39	N/A	95.3 ± 17.5
Yang et al. [37]	2024	China	single-blind, randomized clinical trial	44.7	38.58 ± 10.39	0.942	N/A	77.08 ± 26.51	N/A
				62.8	39.34 ± 12.12	0.832	N/A	82.40 ± 25.17	N/A

N/A-Not available

The majority of studies have focused on IgA nephropathy, with a few addressing membranous nephropathy (Table 2). The most common intervention is hydroxychloroquine (HCQ), often combined with other treatments like RASI (Renin-Angiotensin System Inhibitors (RASI), immunosuppressants (IS), or corticosteroids (CS). Control groups in most studies received either RASI(predominantly losartan) or placebo. The treatment duration typically ranges from 6 to 24 months, with most studies using a 6-month period. Some studies, such as Si 2023, have extended the treatment duration to 24 months. Proteinuria is generally lower in the intervention groups compared to control groups, with the lowest value reported to be 0.22 g/day [31] to 2.5 ± 0.9 g/day [29]. Most studies showed better eGFR in the intervention groups than in the controls. However, GFR values showed variability, ranging from 47.7 ± 25.2 ml/min/1.73 m² [34] to 96.06 ml/min/1.73 m² [32].

**Table 2:** Efficacy of hydroxychloroquine in included studies

References	Sample size	Glomerular disease	Intervention	Treatment duration	Clinical outcomes		
					Proteinuria (g/day)	Serum creatinine (mg/dL)	eGFR (ml/min/ 1.73 m2)
Bagchi et al. [29]	37	IgA Nephropathy	HCQ only	6 months	2.5 ± 0.9 (responders), 1.8 ± 0.4 (non-responders)	-	-
Gao et al. [30]	14	IgA Nephropathy	HCQ + Losartan	6 months	0.5 ± 0.2	89 ± 22	81 ± 21
	14		Losartan (control)	6 months	0.7 ± 0.3	88 ± 18	81 ± 19
He et al. [31]	57	IgA Nephropathy	HCQ + RASI	6 months	0.22	-	77.39 ± 13.48
	50		RASI (control)		0.47	-	80.65 ± 17.03
Liu et al. [18]	30	IgA Nephropathy	HCQ + RASI	6 months	0.9	-	53.1 ± 20.2
	30		RASI (control)		1.9	-	55.2 ± 19.7
Liu et al. [32]	40	IgA Nephropathy	HCQ + RASI	6 months	0.61	80.40	84.78
	40		RASI (control)		0.46	81.90	96.06
Mei et al. [22]	50	PLA2R-associated membranous nephropathy	HCQ + RASI	6 months	1.75	-	81.6 ± 13.4
	45		RASI (control)		2.67	-	80.2 ± 14.1
Si et al. [20]	39	IgA Nephropathy	HCQ only	24 months	0.97	-	66.10 ± 28.00
	39		CS*		0.53	-	67.04 ± 23.80
Tang et al. [33]	26	IgA nephropathy	HCQ + IS**	6 months	1.10	-	50.91
	26		IS (control)		1.24	-	56.39
Tang et al. [19]	180	IgA Nephropathy	HCQ	12 months	1.01	-	63.93 ± 25.96
Yang et al. [34]	90	IgA Nephropathy	HCQ + RASI	6.4 months	0.8	-	47.7 ± 25.2
	90		RASI (control)		1.2	-	50.5 ± 17.6
Yang et al. [35]	92	IgA nephropathy	HCQ	6 months	0.8	-	-
	92		CS (control)		0.7	-	-
Yang et al. [36]	31	Membranous nephropathy	HCQ	6 months	1.2	-	-
	41		Control		2.2	-	-
Yang et al. [37]	38	IgA Nephropathy	HCQ + RASI	6 months	0.542	81.84 ± 26.57	-
	35		RASI (control)		0.883	80.74 ± 22.08	-

*CS- Corticosteroids; RASI-Renin angiotensin system inhibitors

**IS-Immunosuppressants: Cyclophosphamide/Tacrolimus/Mycophenolate mofetil/Leflunomide/Cyclosporine A

### Quality assessment

Three studies were non-randomized cohort studies, scoring above 5 points on the Newcastle- Ottawa scale, suggesting that they were of moderate to good quality (Table 3a). Six non- randomized case-control studies scored 7 points on the Newcastle-Ottawa scale, indicating a high quality (Table 3b). Randomized studies scored between 3 and 5 points on the Cochrane Risk of Bias 2 scale, indicating a medium risk of bias (Table 3c).

**Table 3a:** Quality assessment for non-randomized (cohort) studies

Study		Selection				Comparability		Outcomes			Total
First Author	Year	1	2	3	4	5	6	7	8	9	
Bagchi	2022	*	-	*	*	*	-	*	*	-	6/9
He	2024	*	*	*	*	*	-	*	*	-	7/9
Tang	2022	*	-	*	*	*	-	*	*	-	6/9

1 = Representativeness of exposed cohort; 2 = Selection of non-exposed cohort; 3 = Ascertainment of exposure; 4 = Definition of control/ Outcome not present at study initiation; 5 = Main factor; 6 = Additional factor; 7 = Assessment of outcomes; 8 = Sufficient follow-up time; 9 = Adequacy of follow-up

‘*’ denotes that the study fulfils the criterion whereas ‘-’ denotes that the study did not fulfil the criterion

**Table 3b:** Quality assessment for non-randomized (case-control) studies

Study		Selection					Comparability		Outcomes				Total
First Author	Year	1	2	3	4	5		6		7	8	9	
Gao	2017	*	-	*	*	*		*		*	*	-	7/9
Liu	2022	*	-	*	*	*		*		*	*	-	7/9
Si	2023	*	-	*	*	*		*		*	*	-	7/9
Tang	2020	*	*	*	*	*		-		*	*	-	7/9
Yang	2018	*	*	*	*	*		-		*	*	-	7/9
Yang	2019	*	*	*	*	*		-		*	*	-	7/9

1 = Adequacy of Case Definition; 2 = Representativeness of Cases; 3 = Selection of Controls; 4 = Definition of Controls; 5 = Main factor; 6 = Additional factor; 7 = Ascertainment of Exposure; 8 = Same Method of Ascertainment for Cases and Controls; 9 = Non-Response Rate

‘*’ denotes that the study fulfils the criterion whereas ‘-’ denotes that the study did not fulfil the criterion

**Table 3c:** Quality assessment of randomized studies

Study		1	2	3	4	5	Total
Author	Year						
Yang	2024	-	*	*	*	-	3/5
Yang	2024	*	*	*	*	*	5/5
Liu	2019	*	*	*	*	*	5/5
Mei	2024	*	-	*	*	*	4/5

1 = Bias arising from the randomization process; 2 = Bias due to deviations from intended interventions; 3 = Bias due to missing outcome data; 4 = Bias in measurement of the outcome; 5 = Bias in selection of the reported result

‘*’ denotes that the study fulfils the criterion whereas ‘-’ denotes that the study did not fulfil the criterion

## Effectiveness of HCQ on clinical outcomes

### Impact of HCQ on proteinuria

#### Impact of HCQ on proteinuria based on treatment duration

**Fig. 2:**
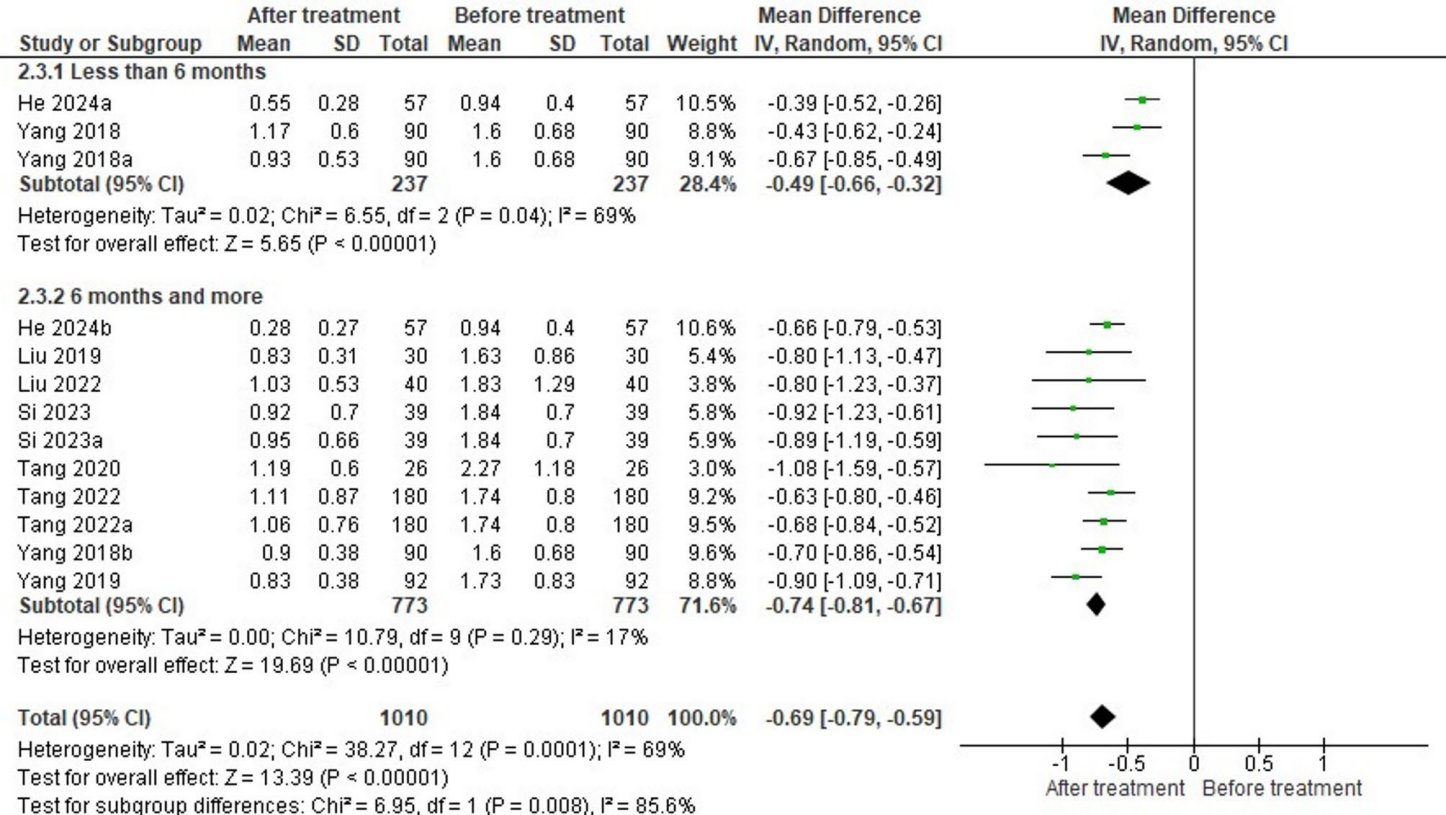
Forest plot on the effectiveness of HCQ on proteinuria level based on treatment duration

Eight studies reported the 24-h urinary protein levels in patients at the end of HCQ treatment. The pooled analysis revealed a significant reduction in proteinuria in 1010 patients following HCQ treatment (MD = -0.69, 95% CI= -0.79, to -0.59, *P* < 0.00001), with moderate heterogeneity observed across the included studies (I^2^ = 85.6%, *P* = 0.008) (Fig. 2).

Subgroup analysis was performed based on the treatment duration. In only two studies, 237 patients were treated with HCQ for less than 6 months (subgroup 1), while the remaining six studies investigated the impact of HCQ on proteinuria in 773 patients when administered for ≥ 6 months (subgroup 2). Subgroup analysis showed a significant difference in proteinuria decrease in patients treated with HCQ for both short-term (MD = -0.49, 95% CI= -0.66 to -0.32, *P* < 0.00001) and long-term (MD = -0.74, 95% CI= -0.81, -0.67, *P* < 0.00001) durations compared to baseline levels. However, the second subgroup showed a stronger and more consistent effect with low heterogeneity (I² = 17%) than the first subgroup (I² = 69%). Overall, HCQ treatment showed a statistically significant and clinically relevant improvement in outcomes, with stronger effects observed for treatment durations of ≥ 6 months.

**Fig. 3:**
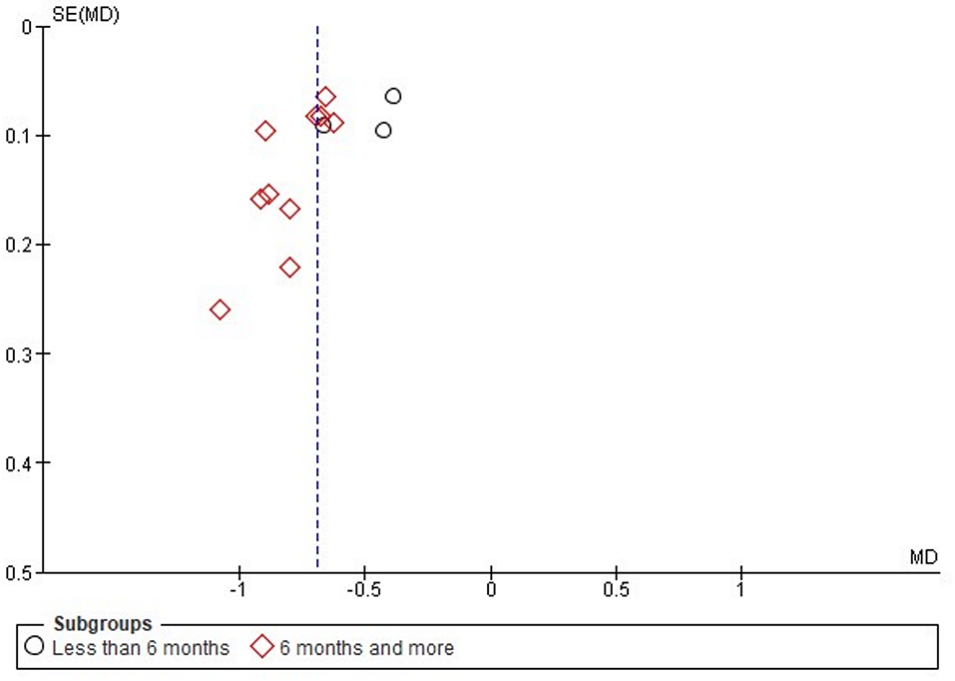
Funnel plot on the effectiveness of HCQ on proteinuria level based on treatment duration

The funnel plot appeared to be symmetrical for subgroup 2 (6 months or more), indicating a lower risk of publication bias (Fig. 3). However, there is a moderate risk of publication bias for slight asymmetry in the first subgroup (less than 6 months), with some points deviating from the triangular shape.

### Impact of HCQ on proteinuria based on disease types

**Fig. 4:**
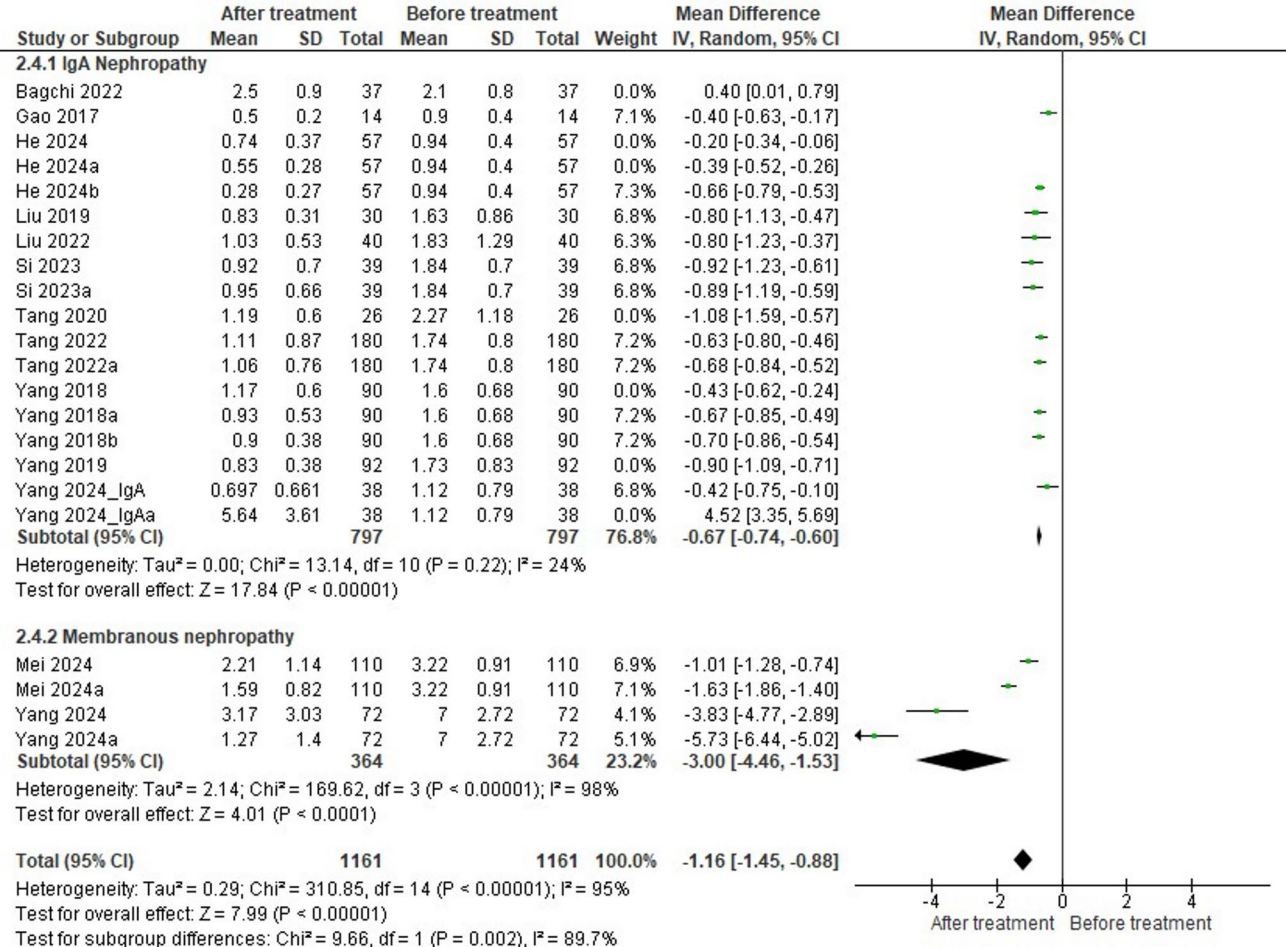
Forest plot on the effectiveness of HCQ on proteinuria level based on disease types

Thirteen studies compared the effects of HCQ treatment on proteinuria levels in 1161 patients with two types of nephropathy (IgA nephropathy versus membranous nephropathy) (Fig. 4). Subgroup analysis showed a significant reduction in the measured parameters for 797 patients with IgA nephropathy following HCQ treatment (MD = -0.67, 95% CI= -0.74 to -0.60, *P* < 0.00001), with low heterogeneity across studies (I^2^ = 24%). On the other hand, there was a more substantial reduction in the parameter for 364 patients with membranous nephropathy compared to IgA nephropathy (MD = -3.00, 95% CI= -4.46 to -1.53, *P* < 0.0001). However, the high heterogeneity (I² = 98%) suggests that the results vary considerably across studies, possibly because of differences in populations, interventions, or measurements. Pooled analysis revealed that treatment was associated with a statistically significant reduction (MD = -1.16, 95% CI= -1.45 to -0.88, *P* < 0.00001). However, the high heterogeneity (I^2^ = 95%) indicated variability across the included studies.

**Fig. 5:**
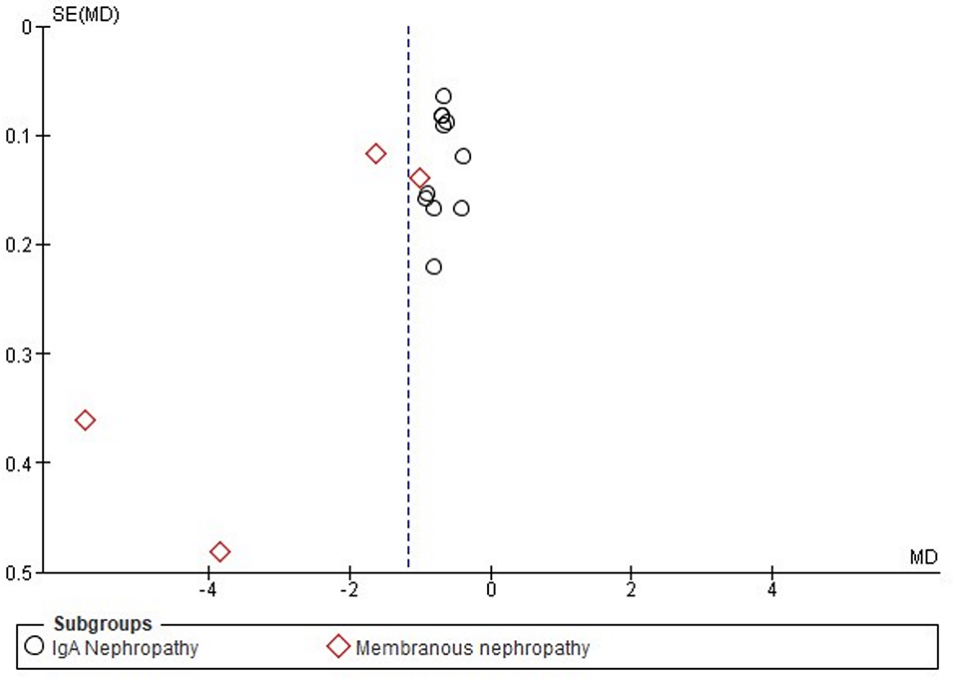
Funnel plot on the effectiveness of HCQ on proteinuria level based on disease types

The funnel plot shows the data for both subgroups, IgA nephropathy (circles) and membranous nephropathy (diamonds) (Fig. 5). Most studies on IgA nephropathy were scattered close to the central line (MD = 0), with no significant outliers. The majority of the studies were clustered near the top, indicating low standard errors. Therefore, the plot was relatively symmetric for IgAN, indicating a lower likelihood of publication bias. However, for membranous nephropathy, some points deviate more significantly from the central line, suggesting possible heterogeneity or publication bias.

### Impact of HCQ on eGFR

### Impact of HCQ on eGFR based on treatment duration

**Fig. 6:**
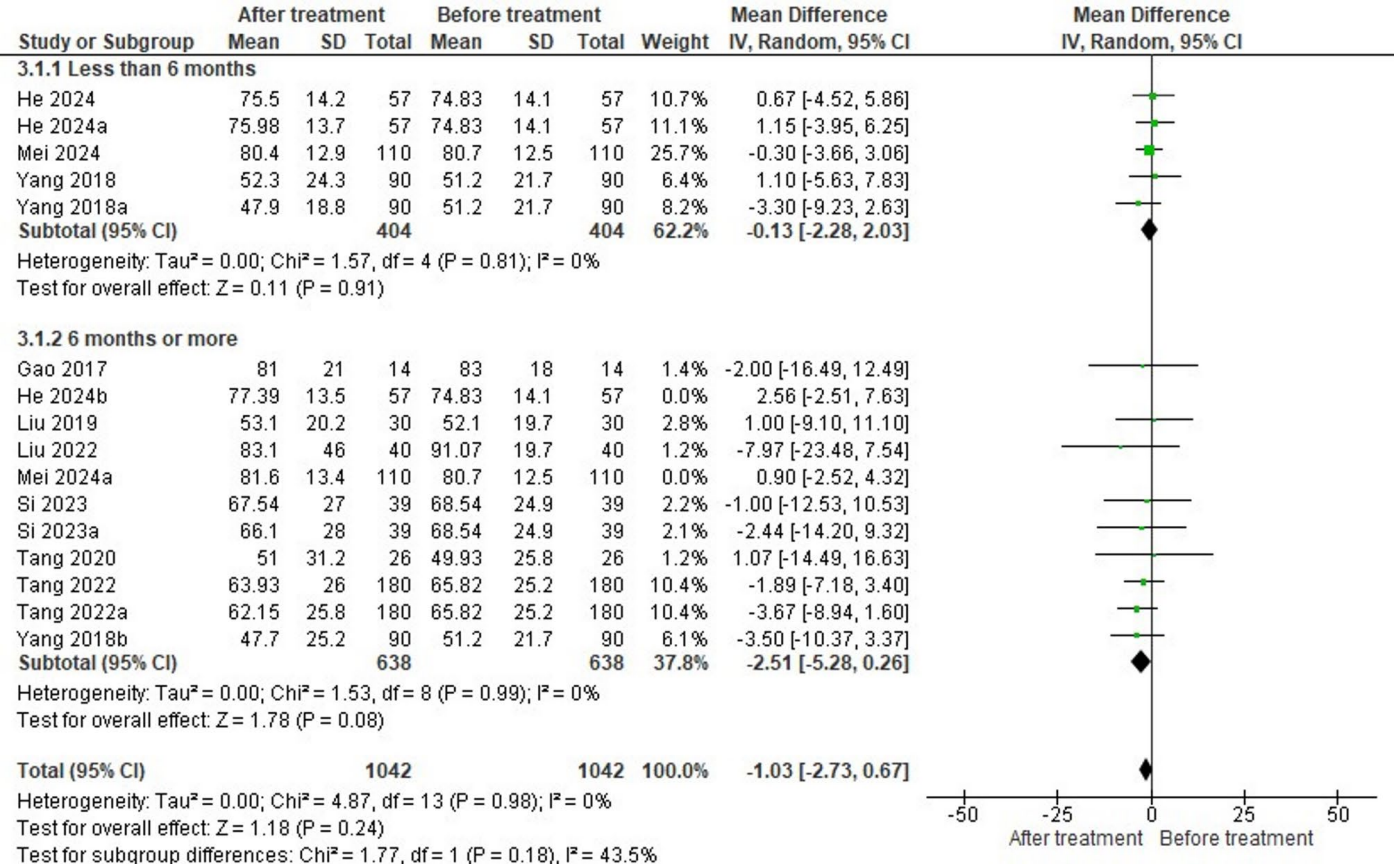
Forest plot on the effectiveness of HCQ on eGFR level based on treatment duration (He 2024a-treatment duration less than 6 months, Yang 2018a-treatment duration less than 6 months, He 2024b-treatment duration more than 6 months, Mei 2024a-treatment duration greater than 6 months, Tang 2022a-treatment duration more than 6 months, Yang 2018b-treatment duration greater than 6 months)

Nine studies reported the eGFR levels in 1042 patients at the end of HCQ treatment. Pooled analysis revealed that there was no significant improvement in eGFR following HCQ treatment (MD = -1.03, 95% CI= -2.73, to 0.67, *P* = 0.24) (Fig. 6). Sub-group analysis was done based on treatment duration. In only three studies, 404 patients were treated with HCQ for less than 6 months (subgroup 1), while the remaining six studies investigated the impact of HCQ on eGFR in 638 patients when administered for ≥ 6 months (subgroup 2). The subgroup analysis also showed no statistically significant improvement in outcomes based on treatment duration (MD for sub-group 1 = -0.13, 95% CI= -2.28 to 2.03, *P* = 0.91, and MD for sub-group 2 = -2.51, 95% CI= -5.28 to 0.26, *P* = 0.08). Heterogeneity was absent for both subgroups (Tau² = 0; I² = 0%), indicating consistency across studies.

**Fig. 7:**
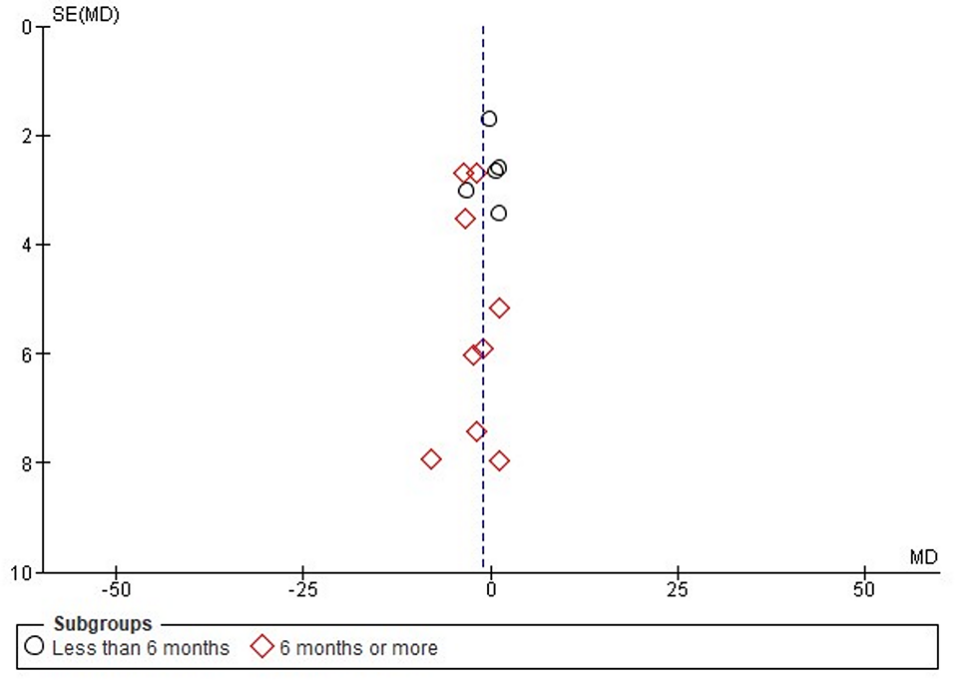
Funnel plot on the effectiveness of HCQ on eGFR level based on treatment duration

The funnel plot appears to be symmetrical (Fig. 7). Studies in both subgroups are scattered close to the central line (MD = 0), with no significant outliers. The majority of studies have small standard errors, indicating high precision. Studies under sub-group 1 (circles) and sub-group 2 (diamonds) are fairly well distributed around the vertical axis, further supporting the low risk of publication bias.

### Impact of HCQ on eGFR based on disease types

**Fig. 8:**
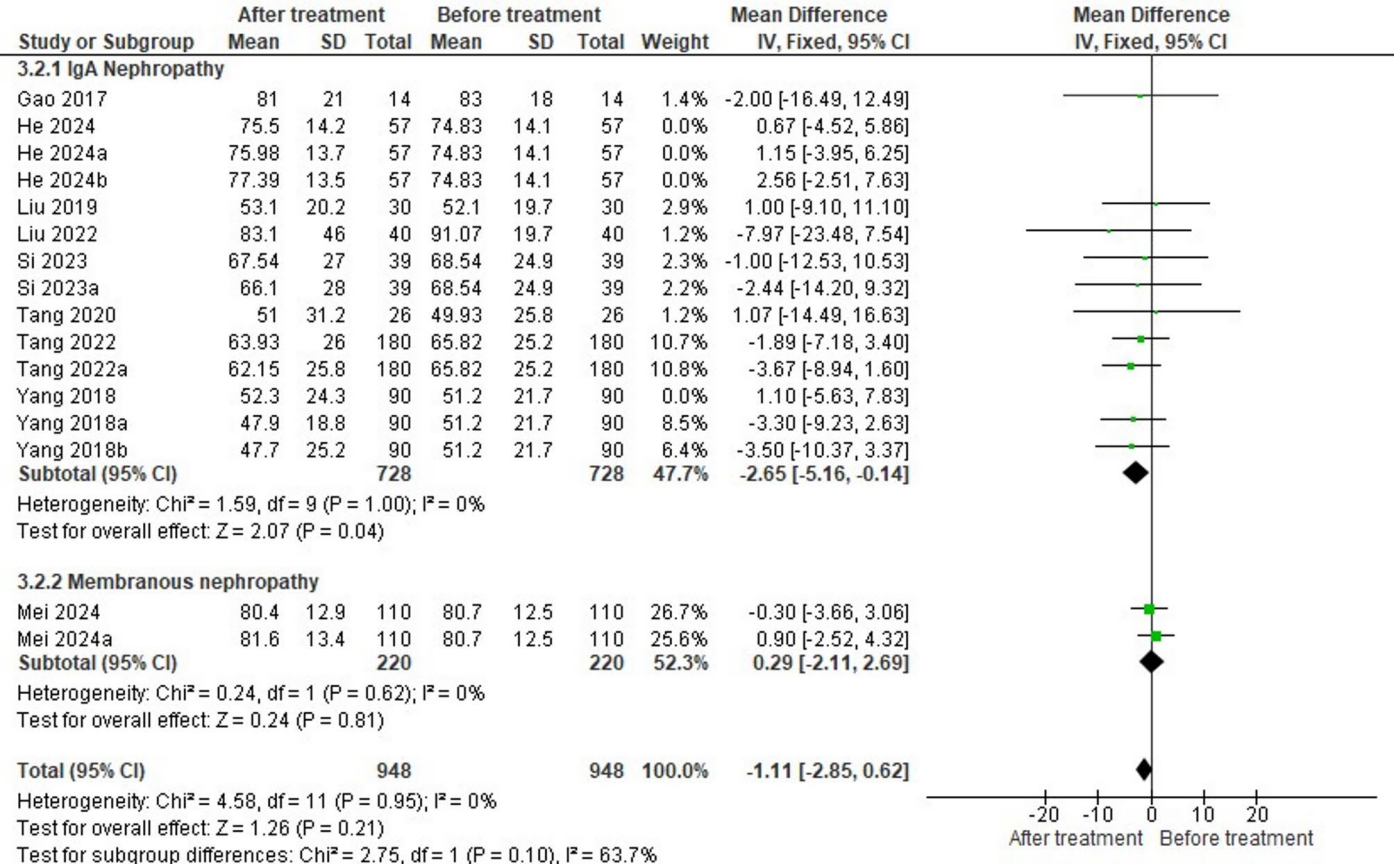
Forest plot on the effectiveness of HCQ on eGFR level based on disease types

Nine studies compared the effects of HCQ treatment on eGFR levels in 948 patients with two types of nephropathy (IgA nephropathy vs. membranous nephropathy) (Fig. 8). Subgroup analysis showed a small but significant reduction in mean values after treatment in 728 patients with IgA nephropathy, suggesting a positive effect of the intervention (MD = -2.65, 95% CI= -5.16 to -0.14, *P* = 0.04). However, the results did not indicate a significant change in the mean values after treatment in 220 patients with membranous nephropathy (MD = 0.29, 95% CI= -2.11, to 2.69, *P* = 0.81). The pooled analysis also revealed no statistically significant effect of treatment on eGFR across all nephropathy types (MD = -1.11, 95% CI= -2.85 to 0.62, *P* = 0.21). For both subgroups and overall, the heterogeneity was absent (Tau² = 0; I² = 0%), indicating consistency across studies.

**Fig. 9:**
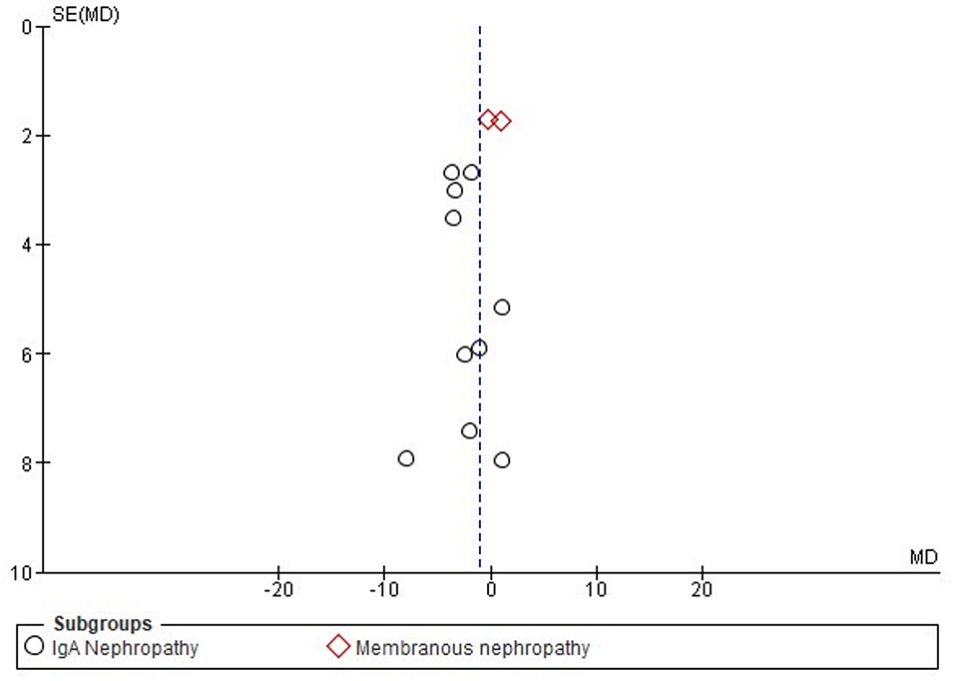
Funnel plot on the effectiveness of HCQ on eGFR level based on disease types

Overall, the funnel plot appears to be symmetrical (Fig. 9). Most studies are scattered close to the central line (MD = 0), with no significant outliers. The majority of studies are clustered near the top, indication low standard errors. All these findings indicate low risk of publication bias. However, more studies are needed for sub-group 2 to assess its funnel symmetry accurately.

### Impact of HCQ on remission and relapse rates

Only three studies have examined the remission and relapse rates of primary glomerular disease associated with HCQ treatment. In Bagchi et al. [29] HCQ supplementation led to remission in 56.8% of patients (27% CR and 29.7% PR) without progression to significant renal failure after six months of treatment. During the 22.0 months median follow-up (range: 7.4–27.6), approximately 22% of patients relapsed after discontinuing HCQ. Of these, 50% achieved remission upon reintroducing HCQ, whereas 37.5% experienced persistent proteinuria, indicating partial efficacy. In Gao et al. [30] HCQ supplementation improved remission rates to 42.9% (21.4% CR and 21.4% PR) compared to 14.3% (only CR) in the control group, demonstrating a significant benefit. Yang et al. [36] reported that treatment with HCQ resulted in higher remission rates at both six months (87.1%) and 12 months (93.5%) compared to the control group (73.2% and 90.2%, respectively). Relapse rates were also lower, with only one patient in the HCQ group relapsing, compared to seven in the control group, after eight months of remission. These findings suggest that HCQ supplementation enhances remission rates and reduces relapse rates in primary glomerular disease, with sustained efficacy in managing relapse when reintroduced.

### Safety profile of HCQ

The safety of HCQ therapy was evaluated by determining the incidence rate and types of adverse effects experienced by patients with primary glomerular diseases. A total of 69 adverse events were reported across the studies included in this review, with varying frequencies for different physiological systems (Table 4). The highest number of adverse events was reported by Mei et al. [22] with 14 cases, followed by Yang et al. [35] with 12 cases and Tang et al. [19] with 10 cases. Gastrointestinal and mucocutaneous effects, each accounting for 20 cases, were the most commonly reported adverse events, representing approximately 58% of the total adverse events. Among the gastrointestinal effects, liver dysfunction was the most frequently reported (nine cases), followed by nausea (seven cases). Other reported effects included diarrhoea, abdominal pain, constipation, and faecal occult blood (one case each). These complications may result from HCQ-induced alterations in the gut microbiota [38]. To mitigate nausea or abdominal discomfort, it is recommended to take HCQ tablets along with a glass of milk or after a meal. However, antacids should be avoided because they interfere with HCQ absorption [26]. Mucocutaneous-related complications of HCQ therapy included pruritus (nine cases), skin pigmentation changes (nine cases), alopecia (three cases), and desquamation (two cases).

Most of these symptoms are often attributed to allergic responses, possibly due to the high affinity of HCQ for the melanin pigment present in the skin [39]. However, these reactions are mild and affected patients are relieved after symptomatic treatment [22]. Blurred vision from retinal toxicity (2 cases), eGFR reduction (3 cases), and cardiovascular or neurological issues (9 cases) were considered the most severe complications associated with HCQ therapy.

Previous research has highlighted that the risk of severe adverse effects increases with prolonged treatment duration, particularly beyond one year [40, 41]. In contrast, most studies included in this review reported HCQ treatment for six months, with doses tailored to patients’ baseline eGFR values. The shorter treatment duration and tailored dosing may account for the low incidence of severe complications observed in the reviewed studies. Given that many serious adverse effects of HCQ often emerge after extended treatment durations [42], periodic follow-ups are essential to fully characterize its safety profile and manage potential long-term complications.

**Table 4:** Adverse events of HCQ in the studies included

Adverse events	1	2	3	4	5	6	7	8	9	10	11	12	13	Total (*n* = 686)
***Gastro-intestinal effects***														
Liver dysfunction	0	0	3	0	0	2	0	0	0	1	1	2	0	**9**
Nausea	0	0	0	1	0	2	1	1	1	0	1	0	0	**7**
Diarrhoea	0	0	0	0	0	0	0	1	0	0	0	0	0	**1**
Abdominal pain	0	0	0	0	0	1	0	0	0	0	0	0	0	**1**
Constipation	0	0	0	0	0	0	0	0	1	0	0	0	0	**1**
Faecal occult blood	0	0	0	0	0	0	0	0	0	0	0	1	0	**1**
***Urogenital effects***														
eGFR reduction	0	0	0	2	0	0	0	0	0	0	1	0	0	**3**
Urinary tract infection	0	0	0	0	0	0	0	0	0	0	0	1	0	**1**
***Cardiovascular and neurological effects***														
Palpitations	0	0	0	1	0	2	1	0	1	1	1	0	0	**7**
Neuropsychiatric	0	0	0	0	0	0	0	0	0	0	0	0	0	**0**
Dizziness	0	0	0	1	0	1	0	0	0	0	0	0	0	**2**
***Mucocutaneous effects***														
Pruritus	0	0	0	1	0	3	0	0	1	2	2	0	0	**9**
Skin pigmentation	0	0	0	1	0	0	2	1	4	0	1	0	0	**9**
Desquamation	0	0	0	0	0	0	0	0	1	0	1	0	0	**2**
Alopecia	0	0	0	0	0	0	0	0	1	1	1	0	0	**3**
***Anaphylactic effects***														
Dyspnoea	0	0	0	0	0	0	0	0	0	1	1	0	0	2
Rashes	0	0	0	1	0	2	0	0	0	1	1	2	0	7
***Ophthalmologic effects***														
Intraocular pressure elevation	0	0	0	0	0	0	0	0	0	1	1	0	0	2
Blurred vision	0	0	0	0	0	1	0	0	0	0	0	1	0	2
**Total**	**0**	**0**	**3**	**8**	**0**	**14**	**4**	**3**	**10**	**8**	**12**	**7**	**0**	**69**

1 = Bagchi 2022 (*n* = 37); 2 = Gao 2017 (*n* = 14); 3 = He 2024 (*n* = 57); 4 = Liu 2019 (*n* = 30); 5 = Liu 2022 (*n* = 40); 6 = Mei 2024 (*n* = 50); 7 = Si 2023 (*n* = 39); 8 = Tang 2020 (*n* = 26); 9 = Tang 2021 (*n* = 180); 10 = Yang 2018 (*n* = 90); 11 = Yang 2019 (*n* = 92); 12 = Yang 2024 (*n* = 31); 13 = Yang 2024 (*n* = 38)

## Discussion

This study aimed to identify the role of hydroxychloroquine as an adjunct to the standard of care for the reduction of proteinuria and preservation of eGFR, and its safety profile was thoroughly investigated. Hydroxychloroquine is an antimalarial and immunomodulator that can reduce proteinuria by modulating the expression of MMP2 (Matrix metalloproteinase 2), IGF1 (Insulin growth factor 1), and peroxisome proliferator-activated receptor gamma (PPARG) via miR-130b-3p (micro-RNA 130b-3p) [43]. HCQ negatively influences miR-130b-3p, thereby ameliorating the expression of MMP2, IGF1and PPARG which are primarily responsible for damage to the glomerulus and renal tubulointerstitium by recruiting inflammatory cells and fibroblasts [43]. This mechanism has been postulated as the primary mode of proteinuria reduction, predominantly in IgA nephropathy and proteinuric glomerular diseases [43].

Our research showed no impact of HCQ on eGFR, irrespective of treatment duration. HCQ is postulated to offer renoprotection by preferential inhibitory effects on autoimmunity, inhibition of Toll-like receptors, and reduction of production of various cytokines such as interleukin-1 (IL-1), tumour necrosis factor-α (TNF-α), and interferon (IFN)-γ by inflammatory cells, and inhibition of T cell and B cell activation by interfering with calcium-dependent signalling [44]. However, the nature of the analysed studies, short duration of follow-up, weak immunosuppressive action compared of HCQ compared to traditional immunosuppressants, and unique pathological mechanisms of the underlying glomerular diseases that are not tackled by HCQ would have resulted in a non-significant impact on eGFR levels.

The clinical remission (complete and partial) rates in the analysed studies on HCQ treatment ranged from 42.9% to-93.5% [29, 30, 36]. Relapses were even fewer in the HCQ arms [36]. These findings substantiate the utility of HCQ as an adjunct treatment for inducing clinical remission along with the standard of care. Since the endpoints of the majority of studies used proteinuria as a marker for clinical remission, HCQ was found to be a significant molecule that enhances clinical remission.

The safety profile of HCQ is justifiable with predominant side effects, including gastrointestinal(liver dysfunction, nausea, and diarrhoea) and mucocutaneous (pruritus, skin pigmentation, and desquamation) [29–36]. The adverse toxicity of HCQ, which is usually observed when the treatment duration extends beyond one year, includes retinal, cardiovascular, and neurological complications (Table 4). Hence, it is prudent to include HCQ in the treatment armamentarium of primary glomerular disease in select patients without contraindications, considering the acceptable safety profile.

### Limitations

The limitations of this systematic review include the lack of homogeneity among the patients included in the analysed studies, such as differences in underlying pathologies (IgA nephropathy, membranous nephropathy, etc.), stage of kidney disease, and concomitant treatments received, which complicates direct extrapolation of the results. In the included studies, patients treated with HCQ also frequently received immunosuppressants, corticosteroids, or renin-angiotensin system blockers (ACE inhibitors/ARBs), which makes it very difficult to evaluate the real therapeutic impact of HCQ alone. Some of the analysed studies had a short follow-up duration, which made it difficult to analyse the long-term side effects of HCQ. The analysed studies predominantly included Asian populations, and it is difficult to extrapolate these results to other ethnicities. Long-term complications, such as cardiovascular complications, retinal toxicity, and any other non-infectious complications with HCQ, could not be assessed due to the short treatment durations of the included studies.

## Conclusion

This meta-analysis evaluated the effects of HCQ treatment on proteinuria and eGFR in patients with primary glomerular diseases. The findings revealed that HCQ was effective in reducing proteinuria levels in patients, irrespective of disease type and treatment duration. However, varying levels of variability were noted across the included studies, which calls for further well-designed studies to confirm these effects and optimize the treatment protocols. Although no overall improvement in eGFR was observed, a positive impact was noted in patients with IgA nephropathy. Publication bias assessment indicated no significant evidence of bias. In contrast, HCQ therapy for primary glomerular diseases appears to have a manageable safety profile, with most adverse effects being mild and resolving with symptomatic treatment. Severe complications, such as retinal toxicity and cardiovascular issues, are rare but may increase with prolonged use beyond six months. Regular monitoring and tailored dosing are essential to minimize risks and ensure safe treatment outcomes.

## Data Availability

The data for substantiating the findings of this study are available with the corresponding author and can be made available on request.
